# Case Report: Nintedaninb May Accelerate Lung Recovery in Critical Coronavirus Disease 2019

**DOI:** 10.3389/fmed.2021.766486

**Published:** 2021-10-28

**Authors:** Cecilia Bussolari, Diego Palumbo, Evgeni Fominsky, Pasquale Nardelli, Rebecca De Lorenzo, Giordano Vitali, Francesco De Cobelli, Patrizia Rovere-Querini, Anna Mara Scandroglio

**Affiliations:** ^1^Vita-Salute San Raffaele University, Milan, Italy; ^2^Unit of Radiology, Istituto di Ricovero e Cura a Carattere Scientifico San Raffaele Hospital, Milan, Italy; ^3^Unit of Anesthesiology and Intensive Care, Istituto di Ricovero e Cura a Carattere Scientifico San Raffaele Hospital, Milan, Italy; ^4^Division of Immunology, Transplantation and Infectious Diseases, Istituto di Ricovero e Cura a Carattere Scientifico San Raffaele Hospital, Milan, Italy

**Keywords:** severe acute respiratory syndrome Coronavirus 2 (SARS-CoV-2), Coronavirus disease (COVID-19), respiratory dysfunction, lung inflammation, lung recovery, antifibrotic therapy

## Abstract

Severe Coronavirus disease 2019 (COVID-19) is characterized by acute respiratory distress syndrome (ARDS) which may lead to long-lasting pulmonary sequelae in the survivors. COVID-19 shares common molecular signatures with interstitial lung diseases (ILDs), including pro-angiogenic and tissue-remodeling mechanisms mediated by vascular endothelial growth factor receptor (VEGF-R), fibroblast growth factor receptor (FGF-R), and platelet-derived growth factor receptor (PDGF-R). Nintedanib mainly targets these factors and is approved for ILDs. Therefore, we administered nintedanib through compassionate use to three patients with COVID-19 pneumonia requiring extra-corporeal membrane-oxygenation (ECMO). Here, we describe our experience in an attempt to explore the role of nintedanib in lung recovery in COVID-19. Three obese patients aged between 42 and 52 years were started on nintedanib due to difficulty in obtaining lung function restoration and weaning from ECMO support following the removal of orotracheal intubation (OTI). Soon after the start of the treatment, systemic inflammation and respiratory function rapidly improved and ECMO support was withdrawn. Serial chest CT scans confirmed the progressive lung amelioration, also reflected by functional tests during follow-up. Nintedanib was well-tolerated by all the three patients at the dosage used for ILDs and continued for 2–3 months based on drug availability. Although caution in interpreting events is required; it is tempting to speculate that nintedanib may have contributed to modulate lung inflammation and remodeling and to sustain lung repair. Altogether, nintedanib appears as a promising agent in patients with severe COVID-19 and delayed respiratory function recovery, for whom molecularly targeted therapies are still lacking. Clinical trials are necessary to confirm our observations.

## Introduction

Since the beginning of the severe acute respiratory syndrome Coronavirus 2 (SARS-CoV-2) pandemics, more than 166 billion people worldwide have been infected and almost 3.5 million died from Coronavirus disease 2019 (COVID-19) (1). SARS-CoV-2 may cause a wide spectrum of clinical manifestations, the respiratory tract being most commonly affected. A sizable proportion of people with COVID-19 pneumonia develops acute respiratory distress syndrome (ARDS) (2), which may lead to death or long-lasting pulmonary alterations in the survivors (3). Data on the follow-up of patients with COVID-19 in the first wave of the pandemic demonstrate persistent respiratory dysfunction, especially in the case of severe disease forms or a need of mechanical ventilation (4–6). The molecular mechanisms that mediate inflammation beyond viral clearance and sustained tissue damage are not fully understood. Molecules involved in angiogenesis and tissue remodeling, such as vascular endothelial growth factor receptor (VEGF-R), fibroblast growth factor receptor (FGF-R), and platelet-derived growth factor receptor (PDGF-R) have been shown to be increased in patients with COVID-19 (7) and it is tempting to speculate that they may contribute to bolster tissue inflammation and injury.

Nintedanib is a multi-targeted antiangiogenic tyrosine-kinase (TK) inhibitor, which binds the intra-cellular ATP-domain of TK transmembrane receptors, inhibiting their autophosphorylation and activation. It has anti-fibrotic and anti-inflammatory properties and mainly targets VEGF-R, FGF-R, and PDGF-R (8, 9). Nintedanib is approved in patients with idiopathic pulmonary fibrosis (IPF) and other interstitial lung diseases (ILDs), due to its proven beneficial effects on the rate of lung function decline and thus on disease progression (10). Since COVID-19 shares molecular signatures with ILD, we hypothesized that nintedanib might be beneficial in patients with COVID-19, who have delayed lung function recovery, by reducing the lung inflammation and hampering the tissue remodeling. With this rationale, during the second wave of the pandemic, we administered nintedanib through compassionate use to three patients with severe COVID-19 pneumonia requiring extra-corporeal membrane-oxygenation (ECMO). Here we describe our experience in a primordial attempt to determine whether nintedanib may accelerate lung recovery in COVID-19, thus modifying the natural history of the disease.

## Case Description

### Case 1

**Figure 1:**
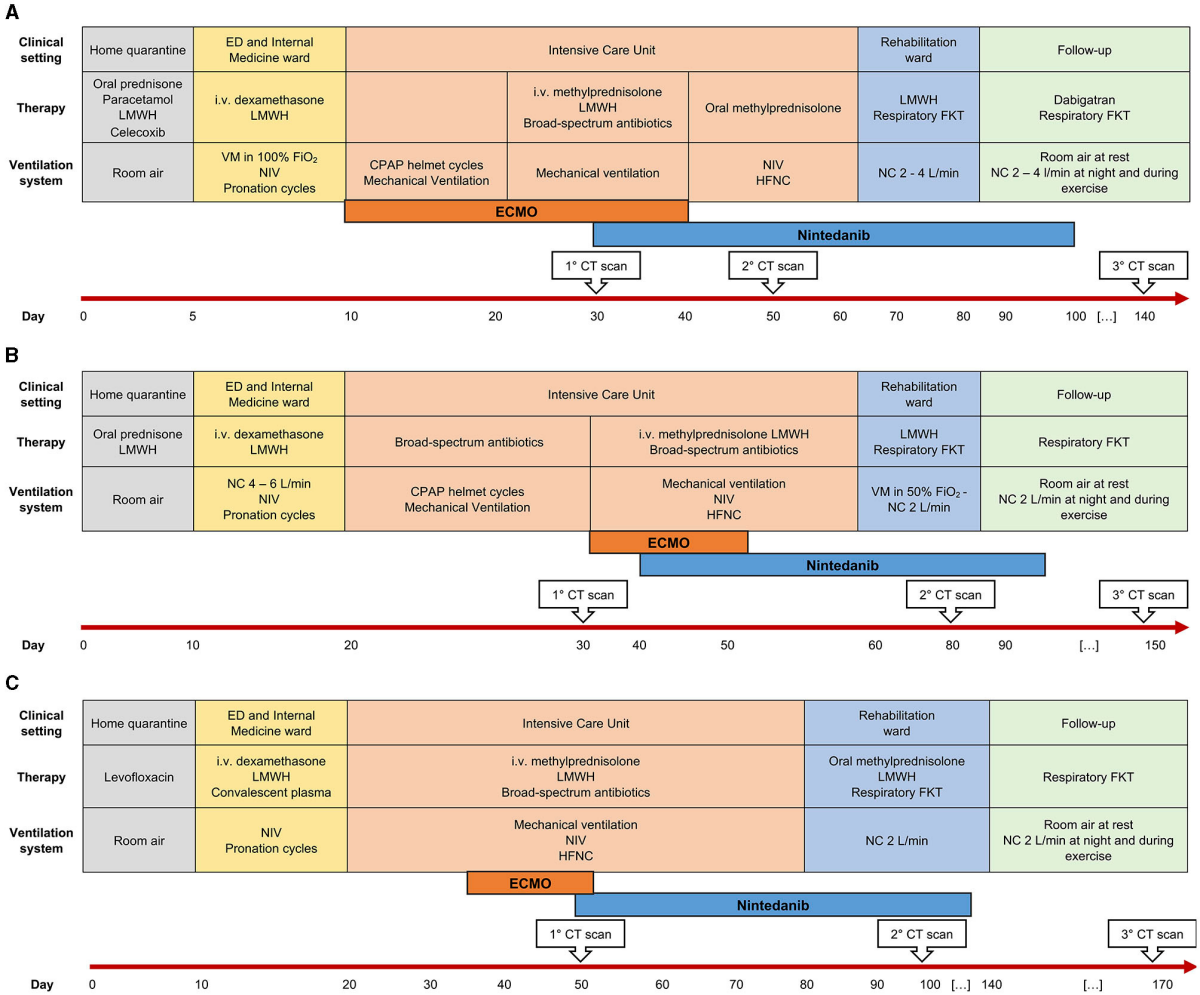
Disease course and patient management over time in three patients **(A–C)**. ED, Emergency Department; LMWH, low-molecular weight heparin; i.v., intravenous; VM, Venturi mask; FiO_2_, fractional inspired oxygen; NIV, non-invasive ventilation; CPAP, continuous positive airway pressure; HFNC, high-flow nasal cannulae; FKT, physiokinesitherapy; NC, nasal cannulae; CT, computed tomography; ECMO, extra-corporeal membrane-oxygenation.

**Figure 2:**
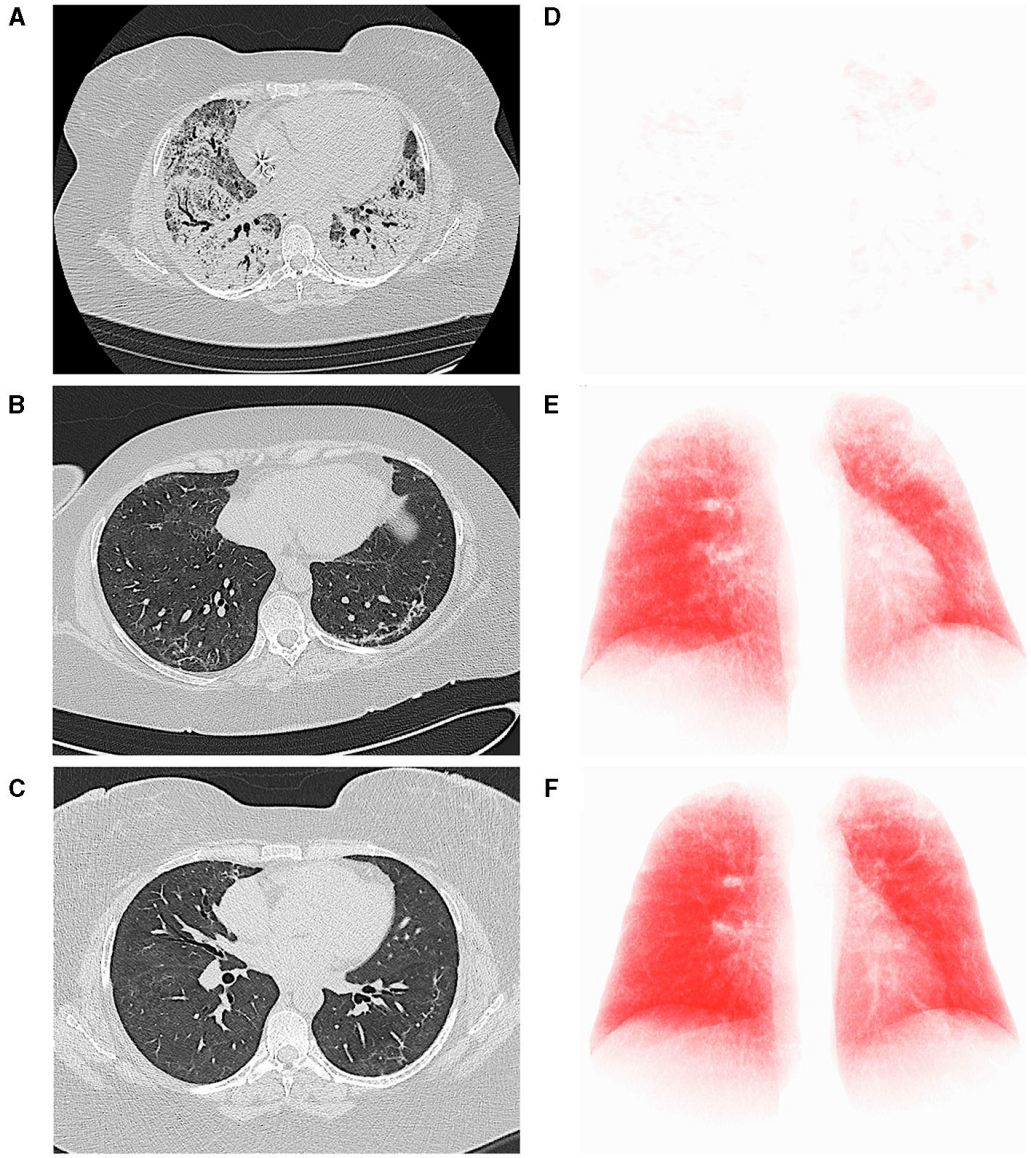
Computed tomography (CT) axial scans (lung parenchyma windowing) acquired, respectively, immediately before **(A)**, 27 **(B)** and 111 days **(C)** after the start of nintedanib, demonstrating progressive improvement of lung parenchyma and significant reduction of residual lung damage. At each time point, the axial CT image most representative of the burden of lung involvement was selected. **(D–F)** report the corresponding three-dimensional volume rendering images (IntelliSpace Portal v.8.0, Philips Medical Systems, Eindhoven, The Netherlands). The red volume represents the well-aerated parenchyma.

**Figure 3:**
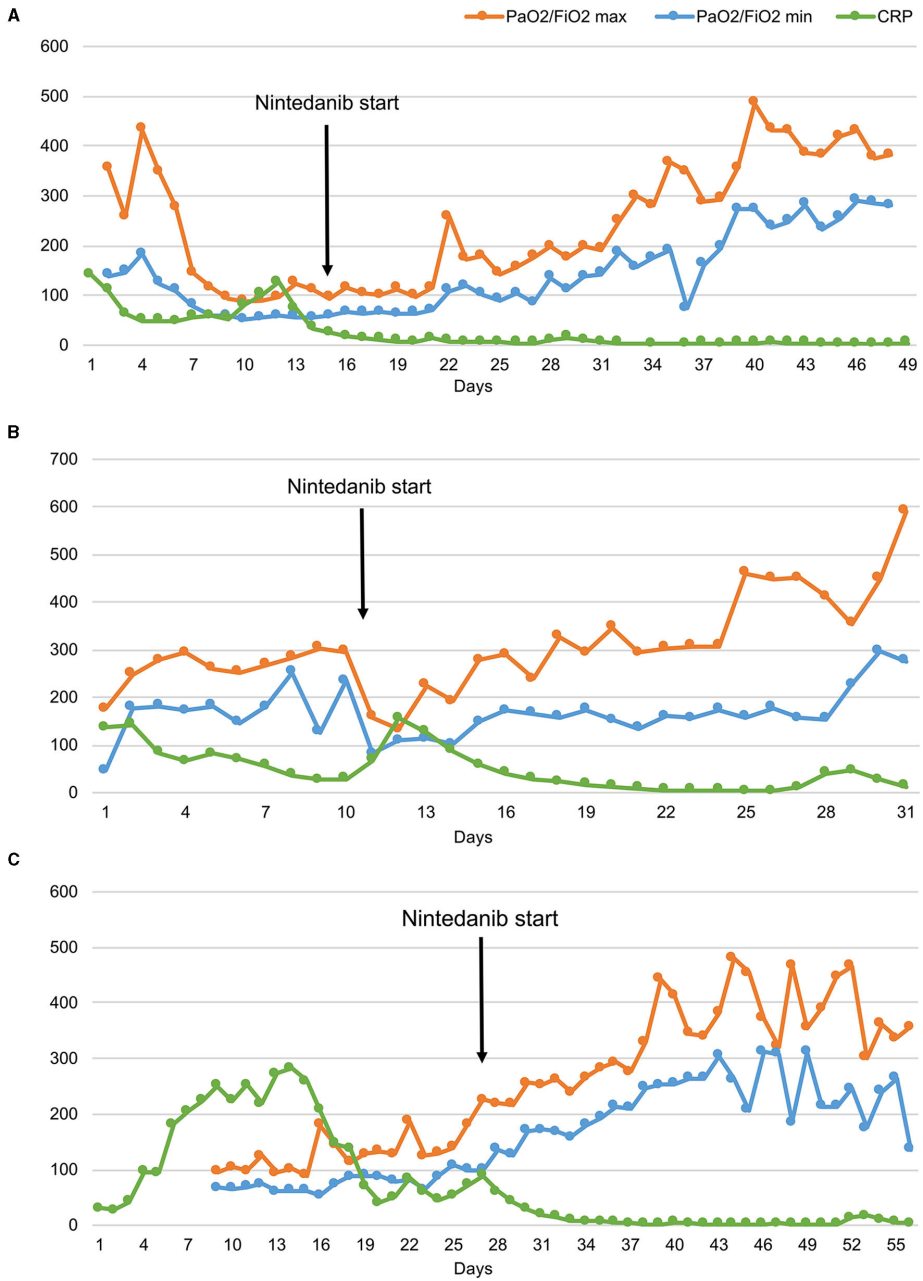
The ratio of partial pressure arterial oxygen to fractional inspired oxygen (PaO_2_/FiO_2)_ and C reactive protein (CRP) levels over time in three patients **(A–C)**. Despite daily variations in minimum and maximum PaO_2_/FiO_2_ values, the trend shows progressive respiratory function improvements since the start of nintedanib in all patients.

CS is a 42-year old woman, obese (BMI 54.11 kg/cm^2^), former smoker of 10 pack-year with silent past medical history except for a previous hospitalization in 2013 for bacterial pneumonia necessitating non-invasive ventilation (NIV) and transfer to the ICU. In November 2020, she developed fever, cough, and diarrhea, and a nasopharyngeal swab for SARS-CoV-2 tested positive. A CT scan showed subcentimetric ground glass opacities (GGOs) in the middle and lower lung lobes. However, she was discharged home with prednisone 1 mg/Kg and subcutaneous low-molecular weight heparin (LMWH) 6,000 UI daily. Two days later, she developed worsening dyspnea and was admitted to the Emergency Department (ED). Arterial blood gas (ABG) analysis showed hypoxemia and high-flow oxygen therapy, continuous positive airway pressure (CPAP), and pronation cycles were started. The patient was hospitalized and intravenous (i.v.) dexamethasone was initiated. Despite therapy, respiratory parameters did not improve and the patient was transferred to the ICU, where orotracheal intubation (OTI) was performed. Due to the need of ECMO support, the patient was then transferred to our tertiary-care hospital. In ICU, she was treated with anticoagulant therapy (bivalirudin, antithrombin), i.v. dexamethasone, and wide-spectrum antibiotic prophylaxis. Due to the progressive stabilization of respiratory function on ECMO support, the patient was extubated and given oral feeding (Figure 1A). On December 4, 2020, a second chest CT scan showed a worsening interstitial thickening, crazy paving areas involving more than 90% of both lungs, and widening of peribronchovascular, mainly subpleural consolidations (Figures 2A,D). In light of the considerable deterioration of the lung tissue, the patient was started on nintedanib at the dosage of 150 mg orally twice a day through compassionate use after obtaining the signature of the informed consent. In the next days, clinical improvement occurred; the ratio of partial pressure arterial oxygen to fractional inspired oxygen (PaO_2_/FiO_2_) rapidly increased and C-reactive protein (CRP) levels were normalized (Figure 3A). Twelve days after nintedanib introduction, the favorable disease course allowed for the suspension of ECMO, while steroid therapy was shifted and tapered to oral methylprednisolone. A chest CT scan performed at day 27 of therapy showed a dramatic improvement in lung parenchymal alterations (Figures 2B,E), with residual subpleural dense bands mainly involving lower lobes.

At day 56 of nintedanib treatment, the patient was discharged from the hospital, although still necessitating oxygen during exercise. Nintedanib was withdrawn after 67 days of therapy. After discharge, the patient underwent serial post-COVID-19 outpatient follow-up evaluations. At one month after ICU discharge (76 days since the start of nintedanib), she still had residual dyspnea and necessitated oxygen therapy with nasal cannulae (NC) at 4 L/min during exercise. Dyspnea disappeared at 3 months (104 days since the start of nintedanib and 37 since its suspension), when she reached 35% of the predicted distance (11) at the 6-minute walking test (6MWT) without any evidence of desaturation on NC at 2 L/min. Chest CT scan performed at this point (day 111 since the start of nintedanib) showed further improvement of lung parenchyma and reduction of residual lung damage (Figures 2C,F). At 6 months (day 158), SpO_2_ at rest was 96% on room air, respiratory rate (RR) 20 breaths/minute, and she walked 43% of the predicted distance without desaturation with minimal oxygen support of 1 L/min (Figure 1A).

### Case 2

MR is a 48-year old, obese (BMI 34.94 kg/cm^2^) man, former smoker of 10 pack-year. He suffers from arterial hypertension, has a family history of dilated cardiomyopathy and sudden cardiac death, and is on chronic therapy with thiazide, angiotensin II receptor blocker and statin. In November 2020, he reported fever, cough, weakness, diarrhea, and dyspnea. A nasopharyngeal swab for SARS-CoV-2 tested positive. The patient was started on oral prednisone 25 mg and LMWH 6,000 UI daily. Few days after symptom onset, he entered the ED for worsening dyspnea. ABG showed hypocapnic hypoxemia and high-flow oxygen therapy and CPAP cycles were started. The patient was hospitalized and i.v., dexamethasone was initiated. Due to persistent reduced PaO_2_/FiO_2_, the patient was transferred to ICU where initial CPAP and prono-supination cycles were soon followed by OTI and mechanical ventilation. Prophylactic antibiotic therapy was administered. Despite therapy, the patient did not show any improvement in the lung function. Chest CT scan showed extensive lung parenchymal alterations with widespread consolidations and signs of initial evolution toward lung fibrosis. The patient was transferred to our third-level hospital to start ECMO support. He was treated with i.v. steroid therapy, anticoagulant therapy (bivalirudin, antithrombin III), and prophylactic i.v. antibiotics. The patient was progressively weaned from ventilator support and finally extubated on December 28. He continued ECMO support and NIV cycles alternated with high-flow O_2_ therapy. As lung function did not improve since extubation, nintedanib was started through compassionate use after obtaining the signature of the informed consent, at the dosage of 150 mg orally twice a day. Eleven days after the introduction of nintedanib, it was possible to discontinue ECMO support due to progressively improved lung function up to PaO_2_/FiO_2_ ratio normalization. The CRP levels decreased concertedly (Figure 3B). The patient was discharged from ICU and transferred to the Rehabilitation Unit. A chest CT scan performed 40 days after nintedanib introduction showed a consistent reduction in consolidating areas, despite the persistence of GGO and peripheral interstitial thickening. At hospital discharge (day 60), the patient was eupneic on room air at rest and necessitated oxygen support with NC at 4 L/min during exercise. A 6MWT on NC at 3 L/min uncovered exertional desaturation (the lowest value of SpO_2_: 78%) and nocturnal oximetry showed episodes of hypopnea necessitating CPAP during sleep. Nintedanib treatment was discontinued after 71 days. A third chest CT scan performed 1 month after nintedanib withdrawal revealed a further reduction of lung alterations, albeit with persisting diffuse GGO. At follow-up evaluation (37 days after nintedanib withdrawal), SpO_2_ at rest was 96% on room air and RR 21 breaths/min. At 6MWT performed with NC at 3 L/min, the patient reached 88% of the predicted distance, with a nadir level of SpO_2_ of 92%. At the following evaluation (81 days after nintedanib suspension), SpO2 at rest was 100% on room air (Figure 1B).

### Case 3

MAP is a 52-year old woman with autoimmune thyroiditis, hypertension, hypercholesterolemia, obesity (BMI 31.51 Kg/cm^2^), and anxiety, on chronic therapy with levothyroxine and statin. In November 2020, she reported nausea, emesis, diarrhea, and lipothymic episodes. A nasopharyngeal swab for SARS-CoV-2 tested positive. Due to the new-onset of dyspnea, the patient was hospitalized, and NIV and prono-supination cycles were initiated. Due to worsening lung function, she was administered convalescent hyperimmune plasma without benefit. She was thus transferred to ICU and intubated. For persisting severe respiratory dysfunction, refractory to NIV and prono-supination cycles, she was transferred to our third-level hospital and started ECMO support. She was treated with anticoagulant therapy (bivalirudin, antithrombin III), i.v. corticosteroid therapy, prophylactic antibiotic therapy, and i.v. anti-hypertensive therapy for blood pressure control. She also necessitated percutaneous tracheostomy for mechanical ventilation. In response to maximal treatment, the respiratory function and general clinical condition of the patient became progressively stable, allowing for OTI removal (Figure 1C). On December 22, 2020, a chest CT scan revealed diffuse bilateral GGO and wide consolidating areas with an air bronchogram at the posterior lower lobes. The same day, the patient was started on nintedanib therapy through compassionate use after obtaining the signature of the informed consent, at the dosage of 150 mg orally twice a day. The PaO_2_/FiO_2_ ratio rapidly improved (Figure 3C), and 6 days after nintedanib introduction, the patient was weaned from the ECMO support. Percutaneous tracheostomy was removed at day 27 of therapy and the patient was transferred to the Rehabilitation Unit. A chest CT scan performed 48 days since nintedanib introduction showed a remarkable reduction of the bilateral parenchymal alterations, with decreased consolidating areas and GGO. At hospital discharge, the patient was eupneic on room air at rest, while still necessitating O_2_ support with NC 2 L/min during exercise. Nintedanib was discontinued after 79 days of therapy. A chest CT scan performed 34 days after drug discontinuation showed the progressive reduction of GGO and consolidating areas, with signs of resolving interstitial pneumonia. At follow-up (83 days after ICU discharge), SpO_2_ was 96% on room air at rest and RR 21 breaths/min. At 6MWT performed on room air, the patient walked 71% of the predicted distance, with a nadir SpO_2_ of 93% (Figure 1C).

## Discussion

Coronavirus disease 2019 is characterized by severe lung alterations which may persist beyond viral clearance and delay pulmonary function recovery (12–15). Here, we describe three cases of patients with COVID-19 requiring orotracheal intubation (OTI) and extra-corporeal membrane-oxygenation (ECMO) support, who were treated with nintedanib due to difficulty in obtaining lung recovery. Nintedanib was administered at the same dosage used in patients with interstitial lung disease (ILD) and the duration of the therapy was established based on the drug availability. Therapy was well-tolerated and no dosage adjustment was necessary. Soon after treatment initiation, a rapid decrease of inflammatory markers was paralleled by a progressive increase in the ratio of partial pressure arterial oxygen to fractional inspired oxygen (PaO_2_/FiO_2)_, allowing for prompt weaning from ECMO support in all patients. Serial chest CT scans confirmed that clinical improvement was reflecting restoration of parenchymal morphology, which progressively ameliorated since the start of nintedanib. The extent to which nintedanib contributed to the observed favorable disease evolution is unknown, prompting caution in interpreting events. However, temporal concordance of nintedanib therapy with the switch toward improvement, as well as the rapidity of respiratory function restoration since drug initiation point to a role of nintedanib in interfering with disease perpetuation and boosting recovery. The cytokine storm underlying the severe COVID-19 has been extensively described (7, 16). Besides T helper (T_H_) 1 and 2 immune response, mediated by several cytokines, such as interleukin (IL)-1β, IL-6, IL-8, IL-12, interferon-γ, IL-2, IL-7, IL-10, and tumor necrosis factor (TNF)–α growth factors also involved in vascular remodeling have been found increased in the lung tissue of patients with COVID-19 (16, 17). Being a small molecule, nintedanib can enter cells and bind several molecular targets, mainly fibroblast growth factor receptor (FGF-R), vascular endothelial growth factor receptor (VEGF-R), and platelet-derived growth factor receptor (PDGF-R). Fibroblast growth factor receptors (FGFs) are produced by macrophages and are mostly involved in angiogenesis and keratinocyte organization (18). Abundant FGF-2 was found in the plasma of patients with COVID-19, regardless of the severity of the illness (7, 17). The VEGF is produced by different cells in response to hypoxia and promote angiogenesis (19). It sustains acute lung injury and acute respiratory distress syndrome (ARDS) through an increased vascular permeability (20). Moreover, it harbors indirect procoagulant properties by altering the hemostatic features of the endothelial cells. The VEGF is increased in the plasma of patients with COVID-19, especially in those developing critical illness, and was recently proposed as a predictor of disease progression (21). The PDGF mediates several biological processes, ranging from angiogenesis to chemotaxis and proliferation of cells (22). It acts as a potent mitogen stimulus for mesenchymal cells, including fibroblasts and smooth muscle cells (22, 23). Similar to FGF, the observed increase in PDGF plasma levels in patients with COVID-19 occurs independent of disease severity (17).

All the above considerations suggest that molecules involved in extracellular matrix regulation and vascular remodeling may contribute to enduring lung damage in patients with COVID-19. Nintedanib, by interfering with this network, could hamper this vicious cycle and promote lung repair. This favorable effect of nintedanib on self-perpetuating inflammation is supported by the striking decrease of systemic inflammation (see data on CPR in Figure 3) and the rapid clinical amelioration that followed drug administration in our patients.

The role of nintedanib appears promising, considering the lack of specific therapies for patients with delayed lung function recovery (2, 24). Clinical trials are necessary to confirm our preliminary findings and to optimize patient selection and drug administration protocols.

## Data Availability Statement

The original contributions presented in the study are included in the article/supplementary material, further inquiries can be directed to the corresponding author.

## Ethics Statement

Written informed consent was obtained from the individual(s) for the publication of any potentially identifiable images or data included in this article.

## Author Contributions

CB and DP: conception and design, acquisition of data, and drafting of manuscript. EF, PN, and GV: acquisition of data. RD: conception and design and drafting of manuscript. FD: acquisition of data and supervision. PR-Q: conception and design, drafting of the manuscript, and supervision. AS: conception and design, acquisition of data, drafting of the manuscript, and supervision. All authors contributed to the article and approved the submitted version.

## Funding

This study was financially supported by Ministero della Salute, Italy, and by COVID-19 donations.

## Conflict of Interest

The authors declare that the research was conducted in the absence of any commercial or financial relationships that could be construed as a potential conflict of interest.

## Publisher's Note

All claims expressed in this article are solely those of the authors and do not necessarily represent those of their affiliated organizations, or those of the publisher, the editors and the reviewers. Any product that may be evaluated in this article, or claim that may be made by its manufacturer, is not guaranteed or endorsed by the publisher.
